# Combination immunotherapy with two attenuated *Listeria* strains carrying shuffled HPV-16 E6E7 protein causes tumor regression in a mouse tumor model

**DOI:** 10.1038/s41598-021-92875-9

**Published:** 2021-06-28

**Authors:** Lin Su, Yunwen Zhang, Xiang Zhang, Ting Liu, Sijing Liu, Yongyu Li, Mingjuan Jiang, Tian Tang, Haiqian Shen, Chuan Wang

**Affiliations:** 1 West China School of Public Health and West China Fourth Hospital, Sichuan University, 17#, Section 3, Renmin Nan Road, Chengdu, 610041 Sichuan People’s Republic of China; 2 Food Safety Monitoring and Risk Assessment Key Laboratory of Sichuan Province, West China School of Public Health, Sichuan University, Chengdu, People’s Republic of China; 3 Nanjing Sungyee Biotechnology Co., Ltd., Nanjing, People’s Republic of China

**Keywords:** Cancer, Cancer therapy, Cancer immunotherapy

## Abstract

Cervical cancer continues to impose a heavy burden worldwide, and human papilloma virus (HPV) infection, especially persistent infection with type 16 (HPV-16), is known to be the primary etiological factor. Therapeutic vaccines are urgently needed because prophylactic vaccines are ineffective at clearing pre-existing HPV infection. Here, two recombinant *Listeria* strains (LMΔ-E6E7 & LIΔ-E6E7) with deletions of the *actA* and *plcB* genes, expressing the shuffled HPV-16 E6E7 protein were constructed. The strains were delivered into the spleen and liver by intravenous inoculation, induced antigen-specific cellular immunity and were eliminated completely from the internal organs several days later. Intravenously treating with single strain for three times, or with both strains alternately for three times significantly reduced the tumor size and prolonged the survival time of model mice. Combination immunotherapy with two strains seemed more effective than immunotherapy with single strain in that it enhanced the survival of the mice, and the LMΔ-E6E7-prime-LIΔ-E6E7-boost strategy showed significant stronger efficacy than single treatment with the LIΔ-E6E7 strain. The antitumor effect of this treatment might due to its ability to increase the proportion of CD8^+^ T cells and reduce the proportion of T regulatory cells (Tregs) in the intratumoral milieu. This is the first report regarding *Listeria ivanovii*-based therapeutic vaccine candidate against cervical cancer. Most importantly we are the first to confirm that combination therapy with two different recombinant *Listeria* strains has a more satisfactory antitumor effect than administration of a single strain. Thus, we propose a novel prime-boost treatment strategy.

## Introduction

Globally, cervical cancer is the fourth-most common cancer in females, it causes almost 266,000 deaths, and there are 528,000 new cases in women each year. Among all the new cases worldwide, 85% occur in the low- and middle-income countries^1^. Persistent infection with specific high risk human papilloma virus (HPV) types is associated with over 99% of cervical cancer cases. Among the high-risk types, HPV type 16 (HPV-16) has the greatest potential to induce cancer and has the highest rate of being detected in women with HPV infection^2^.

Although the currently licensed prophylactic HPV vaccines have shown good effects in terms of preventing preinvasive and invasive cancer, they are ineffective at clearing pre-existing HPV infection^3^. Moreover, incomplete vaccine coverage or the acquisition of HPV infection before prophylactic vaccination leads to a persistent high-risk-type HPV infection and/or HPV-associated lesions in many women. Current treatments for cervical cancer mostly rely on radical surgery, chemoradiation or a combination of these options. With these treatments, 50% of cervical cancer patients succumb to the disease annually^4^. As such, it is important to develop a novel therapeutic regimen against cervical cancer such as immunotherapy. Therapeutic cancer vaccine has been regarded as an innovative immunotherapy that can stimulate cell-mediated immune response targeting tumor-specific antigens, thus retarding tumor growth or curing the cancer^5^. HPV is a double-stranded DNA virus that maintains oncogenic potentiality primarily through the actions of its oncoproteins E6 and E7. The oncoproteins E6 and E7 can disrupt the functions of the tumor suppressor proteins p53 and pRb respectively, and they are constitutively expressed and presented in virus transformed cells^6^. Thus, E6 and E7 are regarded as ideal targets for developing HPV therapeutic vaccines.

To data, several types of HPV therapeutic vaccines have been developed and tested in preclinical and clinical trials, including proteins or peptides vaccines, nucleic acids vaccines, and live vectors vaccines^7^. Live bacterial vector vaccines can induce strong cellular and humoral immune responses because of their strong immunogenicity. *Listeria monocytogenes* (LM) is a promising vaccine vector because it is able to enter either phagocytic or nonphagocytic cells and escape from the phagosome^8^, which allows it to exist in both the cytoplasm and endosomal compartments. Thus the tumor-specific antigen carried by *Listeria* can be presented through major histocompatibility complex (MHC) class I and MHC class II pathway to boost CD8^+^ T cell and CD4^+^ T cell immune responses^9^. *Listeria ivanovii* (LI) is nonpathogenic to humans and has an intracellular life cycle similar to that of LM^10^. LI-based vaccines that used LI as the live vector to deliver antigens are able to induce an antigen-specific CD8^+^ T cell immune response^11^. Therefore, our research utilized both LM and LI as vaccine vectors. Most therapeutic vaccines against cervical cancer provoke immune responses targeting the E7 antigen and some LM-based therapeutic vaccines that target HPV-16 E7 antigen have been developed in previous studies^12,13^. However, one study indicated that DNA vaccines delivering HPV-16 E6E7 have better antitumor potential than those delivering a single antigen^14^. Thus, we used a fusion antigen protein combining E6 and E7 as a tumor target protein in this work. Moreover, all of the previous studies on *Listeria*-based therapeutic vaccines utilized only LM as an antigen delivery carrier, and in nearly all reports, the same LM-based vaccine was applied repeatedly; that is homologous prime-boost immunization was performed. One drawback of homologous prime-boost immunization is that may result in anti-vector immunity, leading to early bacterial clearance, loss of gene expression and limitation of amplification of cellular immune responses^15^. To attempt to overcome this anti-vector immunity, we innovatively utilized both attenuated LM and LI strains in which the two virulence genes *actA* and *plcB* were knocked out to deliver the fused HPV-16 E6E7 antigen protein. Tumor-bearing mice were treated with these two recombinant strains in a prime-boost manner in this study.

In this study, two attenuated recombinant *Listeria* strains named LMΔ*actAplcB-*E6E7 (LMΔ-E6E7) and LIΔ*actAplcB-*E6E7 (LIΔ-E6E7) whose genomes integrated with the selected shuffled HPV16 *E6E7* gene to express and secrete a HPV16 *E6E7* fusion protein were constructed. Then, the 50% lethal dose (LD_50_), bacterial load after intravenous inoculation, antigen-specific cellular response and antitumor effects were tested in a mouse model. We demonstrated the therapeutic efficacy of these strains against cervical cancer by monitoring the tumor size and survival time of tumor-bearing mice. Additionally, we evaluated the proportion of T regulatory cells (Tregs) and CD8^+^ T cells in the intratumoral milieu. This is the first report on a *Listeria ivanovii*-based therapeutic vaccine candidate for cervical cancer. Most importantly, we are the first to confirm that combination therapy with two different recombinant *Listeria* strains has a more satisfactory antitumor effect induced than administration of a single strain. Thus, we propose a novel prime-boost treatment strategy.

## Results

### Construction of LMΔ-E6E7 and LIΔ-E6E7

**Figure 1:**
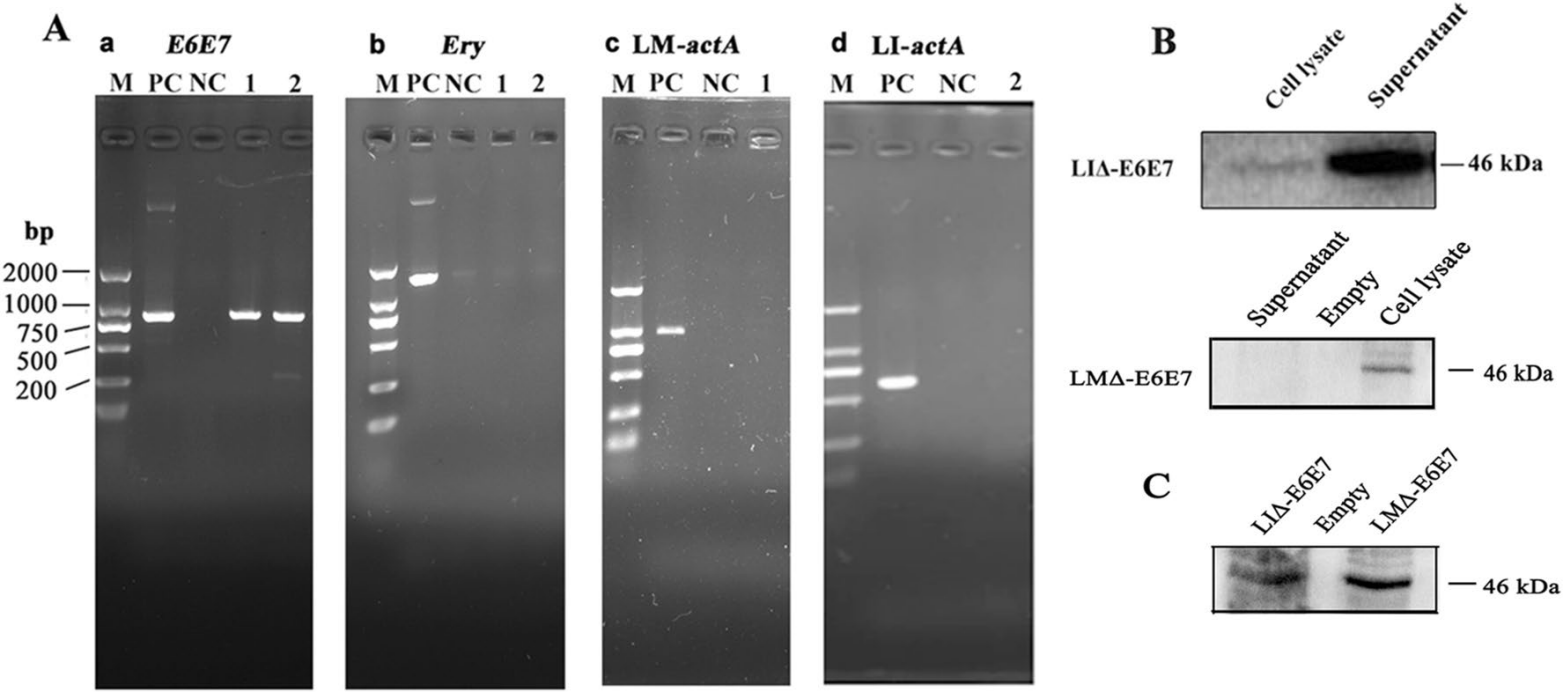
Protein and gene characterization of the recombinant LM and LI strains. (**A**) Genome PCR of the recombinant strains. (a) Amplification of *E6E7* gene from LMΔ-E6E7 (1), LIΔ-E6E7 (2), negative control (NC) and positive control (PC). (b) Amplification of erythromycin resistance gene from LMΔ-E6E7 (1), LIΔ-E6E7 (2), negative control (NC) and positive control (PC). (c) Amplification of LM-*actA* gene from LMΔ-E6E7 (1), negative control (NC) and positive control (PC). (d) Amplification of LI-*actA* gene from LIΔ-E6E7 (2), negative control (NC) and positive control (PC). (**B**) Western blot of culture supernatant and cell lysate from the recombinant strains using the anti-HA monoclonal antibody as primary antibody. (**C**) Western blot of the supernatant of RAW264.7 cell lysate. The RAW264.7 cells were infected with both recombinant strains for 6 h individually, then supernatant of cell lysate was collected for Western blotting. Full-length blots/gels are presented in “Supplementary Fig. S1”. For 1B (LMΔ-E6E7) and 1C, after transferring, according to the protein molecular weight marker, the membranes containing 40–55 kDa protein bands was cut out for incubating with antibodies.

LMΔ-E6E7 and LIΔ-E6E7 were successfully constructed by homologous recombination techniques as described in our previous publication^16^. Erythromycin-sensitive white colonies were considered positive colonies, and suspicious colonies were confirmed by genome polymerase chain reaction (PCR) and sequencing. The results of genome PCR are presented in Fig. 1A. As shown in Fig. 1A, the *E6E7* gene was amplified from the genome of the recombinant strains, indicating that the gene had been successfully integrated into the genome. The erythromycin resistance gene was undetected in the strains, showing that the targeting plasmids had resolved after sequential homologous recombination. The *actA* gene was undetectable, confirming that the recombinant strains were attenuated. The expression and secretion of the fusion antigen protein in the recombinant strains was verified by Western blot analysis. In Fig. 1B, the targeted 46 kD fusion protein was detected in both supernatant and cell lysate from LIΔ-E6E7 or the cell lysate from LMΔ-E6E7. The results indicated that the fusion protein was successfully expressed in the recombinant strains. To explore whether the fusion protein was expressed in vivo, RAW264.7 cells were infected with both strains for 6 h individually, and then cell lysate supernatant was collected for Western blotting. The appearance of the target band indicated that the fusion protein was successfully expressed by cells infected with each recombinant strain (Fig. 1C)*.*

### Virulence of the recombinant strains is reduced in vivo

**Figure 2:**
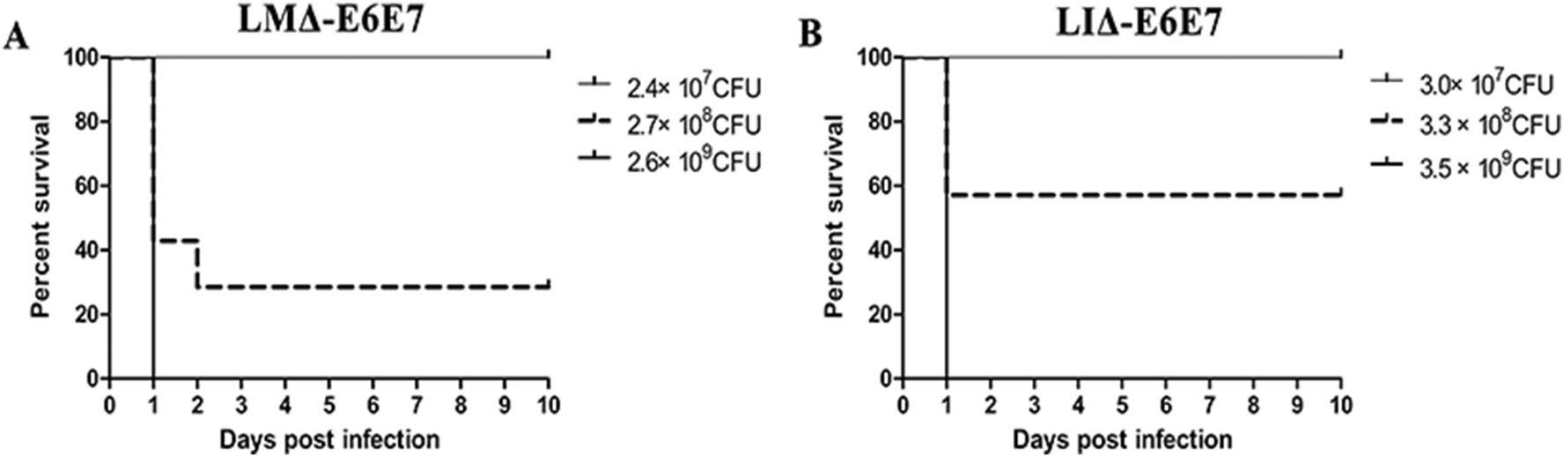
The survival of mice after intravenous injection with the recombinant strain. C57BL/6J mice (7 mice/group) were injected with a gradient-increased dose of LMΔ-E6E7 (**A**) or LIΔ-E6E7 (**B**). Then the mice were monitored for the next 10 days. Experiments were carried out at least three times.

To determine the virulence of LMΔ-E6E7 and LIΔ-E6E7 in C57BL/6J mice, the LD_50_ value was calculated, and the survival curve of each strain is shown in Fig. 2. The LD_50_ value of LMΔ-E6E7 was 1.3 × 10^8^ colony-forming units (CFUs) per mouse, and the LIΔ-E6E7 was 4 × 10^8^ CFUs per mouse. Comparing the LD_50_ values of these strains with the LD_50_ values of the attenuated vector strains previously determined by our laboratory (5 × 10^7^ CFUs per mouse for LMΔ*-lacZ*, 2 × 10^8^ CFUs per mouse for LIΔ*-lacZ*), revealed that both recombinant strains showed decreased virulence in mice.

### Bacterial load of the recombinant strains in target organs after intravenous infection of the mice

**Figure 3:**
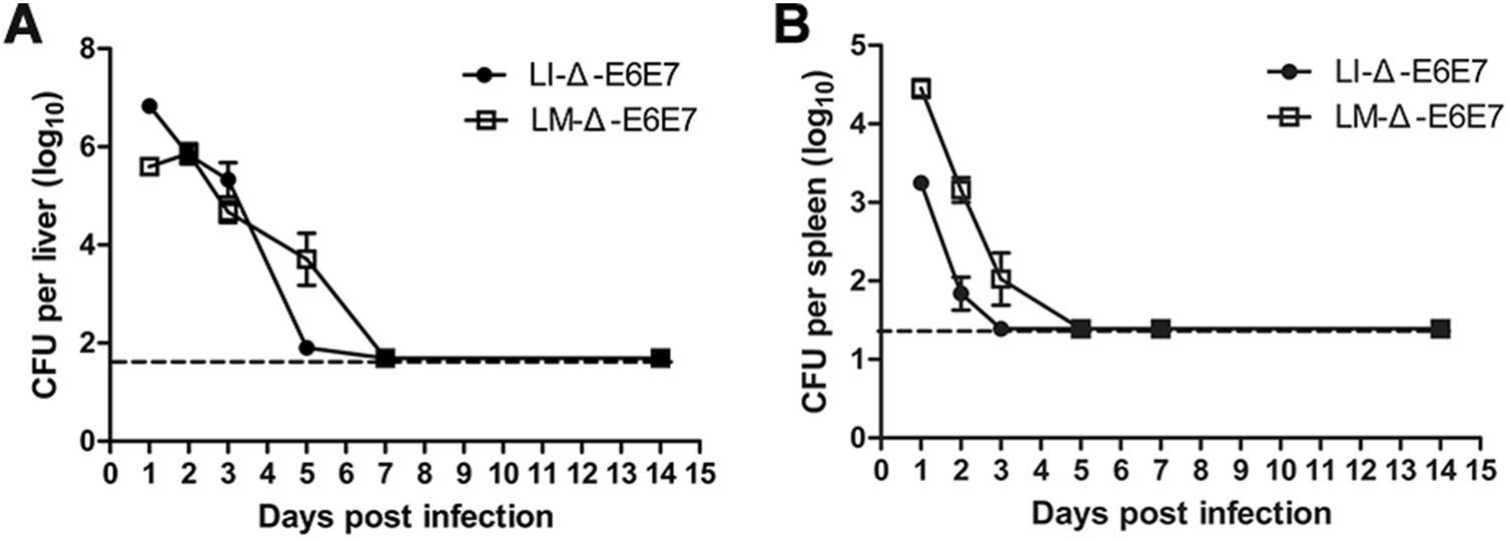
Bacterial load in the liver and spleen after intravenous immunization. Mice were intravenous injected with 1.3 × 10^7^ CFUs LMΔ-E6E7 or 4 × 10^7^ CFUs LIΔ-E6E7, and the bacterial load in the liver (**A**) and spleen (**B**) were determined on 1, 2, 3, 5, 7, 14 dpi. Results present as mean ± SEM per group of five mice from three independent experiments. The dashed line represents the detection limit in each experiment. Experiments were performed in triplicate.

The bacterial loads in the mouse liver and spleen post inoculation of LMΔ-E6E7 or LIΔ-E6E7 are shown in Fig. 3. The bacterial load of LMΔ-E6E7 in the liver was 10^5^ CFUs at 1 day post inoculation (dpi), and then the load maintained or even slightly increased to nearly 10^6^ CFUs at 2 dpi (Fig. 3A). In contrast, the majority of LIΔ-E6E7 entered the liver, causing the bacterial load in the liver to reach 10^6^ CFUs, which was much greater than that of LMΔ-E6E7 on 1 dpi; afterwards, the bacterial load decreased gradually (Fig. 3A). Analysis of the bacterial load in the liver, showed that LIΔ-E6E7 had a better ability to localize in the liver than LMΔ-E6E7. As shown in Fig. 3B, at 1 dpi in the spleen, LMΔ-E6E7 load was 10^4^ CFUs and LIΔ-E6E7 load was 10^3^ CFUs, then the bacterial loads decreased. In addition, the LIΔ-E6E7 load in the spleen became undetectable on 3 dpi, while that of LMΔ-E6E7 decreased to under the detection limit on 5 dpi, showing that LMΔ-E6E7 was inclined to localize to the spleen and had a longer survival time in the host spleen compared with LIΔ-E6E7. Following the intravenous inoculation of the mice, the recombinant strains entered the liver and spleen and survived for a few days. Comparing the loads in the liver and spleen, revealed that both strains seemed to be more inclined to localize to the liver than the spleen. The bacteria in the liver and spleen were completely eliminated and did not resuscitate until 14 dpi, indicating that they are safe for intravenous immunization.

### Recombinant strains elicit strong cellular immunity

**Figure 4:**
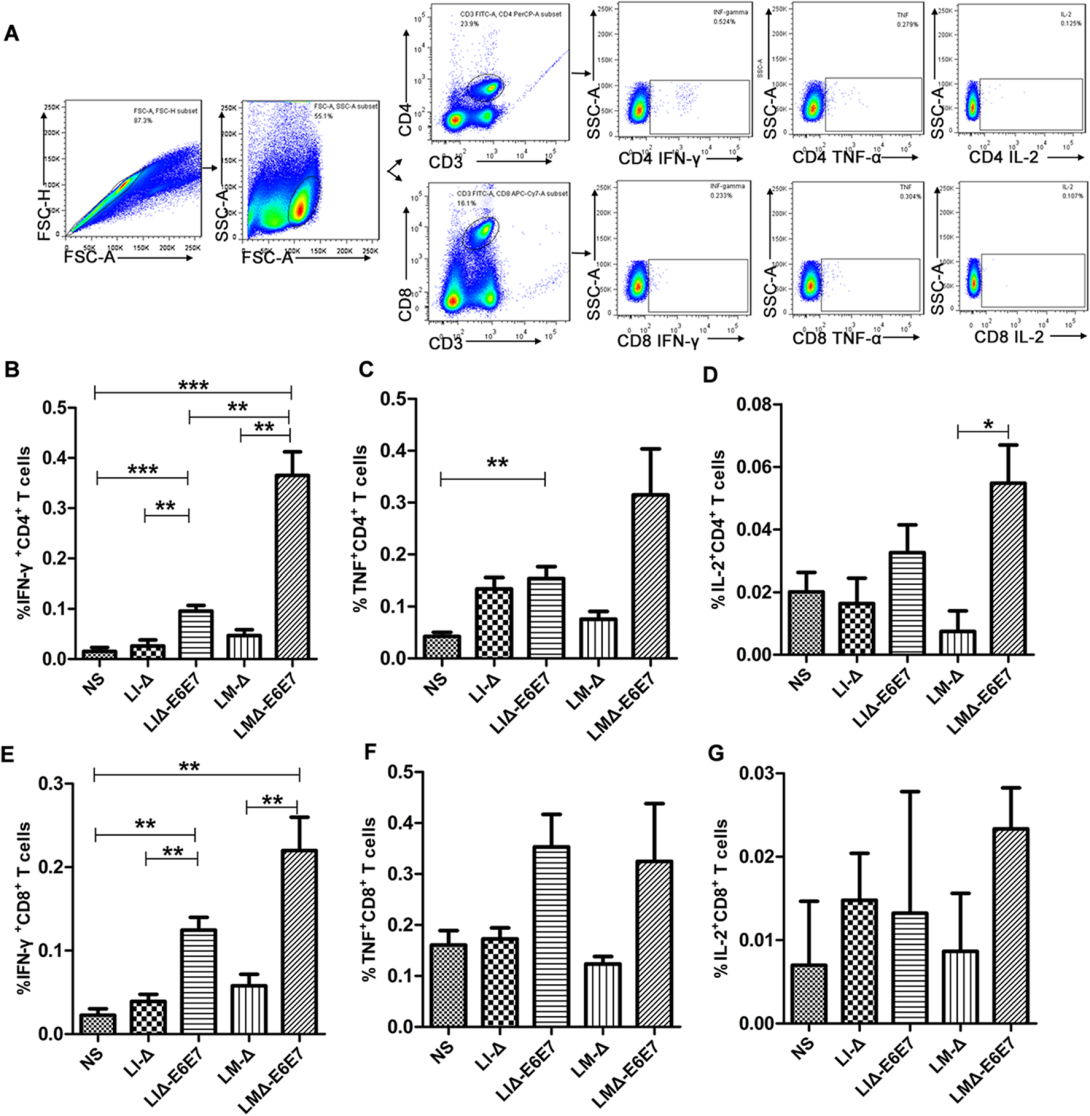
Specific cellular responses induced by the recombinant strains. C57BL/6J mice (9 mice/group) were intravenously immunized with the LMΔ-E6E7, LIΔ-E6E7, LMΔ-*LacZ* and LIΔ*-LacZ* at the dose of 0.1 × LD_50_ or NS as placebo, respectively. Splenocytes were prepared on day 9 after first immunization of vaccines, stimulated with the mixed peptides and detected the percentages of IFN-γ, TNF-α or IL-2 producing CD4 + T and CD8 + T cells by flow cytometry. (**A**) The gating strategy for flow cytometry. (**B**–**G**) Data on the percentages of IFN-γ, TNF-α or IL-2 producing CD4 + T and CD8 + T cells induced by different recombinant strains with significant differences indicated. The data presented (mean ± SEM) were from one independent experiment. All the experiments were performed in triplicate. ***P < 0.001, **P < 0.01, *P < 0.05.

To assess whether the recombinant strains could induce a specific cellular immune response, C57BL/6J mice were intravenously immunized with the recombinant strains, vector strains or normal saline (NS). Intracellular staining of splenocytes was conducted and analyzed by flow cytometry at 9 dpi (Fig. 4). The results indicated that the percentages of IFN-γ-producing CD4^+^ and CD8^+^ T cells were significantly increased in the groups immunized with recombinant strains (LIΔ-E6E7 and LMΔ-E6E7) compared with the control groups (*P* < 0.01) (Fig. 4B,E). Moreover, the percentage of IFN-γ-producing CD4^+^ T cells in mice immunized with LMΔ-E6E7 was higher than that in mice immunized with LIΔ-E6E7 (Fig. 4B). However, there was no significant difference between the percentages of TNF-α-producing CD4^+^ and CD8^+^ T cells and IL-2-producing CD4^+^ and CD8^+^ T cells between the immunized groups and the control groups (Fig. 4C,D,F,G). These results suggested that the recombinant strains induced CD4^+^ and CD8^+^ T cell responses, especially inciting IFN-γ secretion.

### Treatment with recombinant strains induces tumor regression

**Figure 5:**
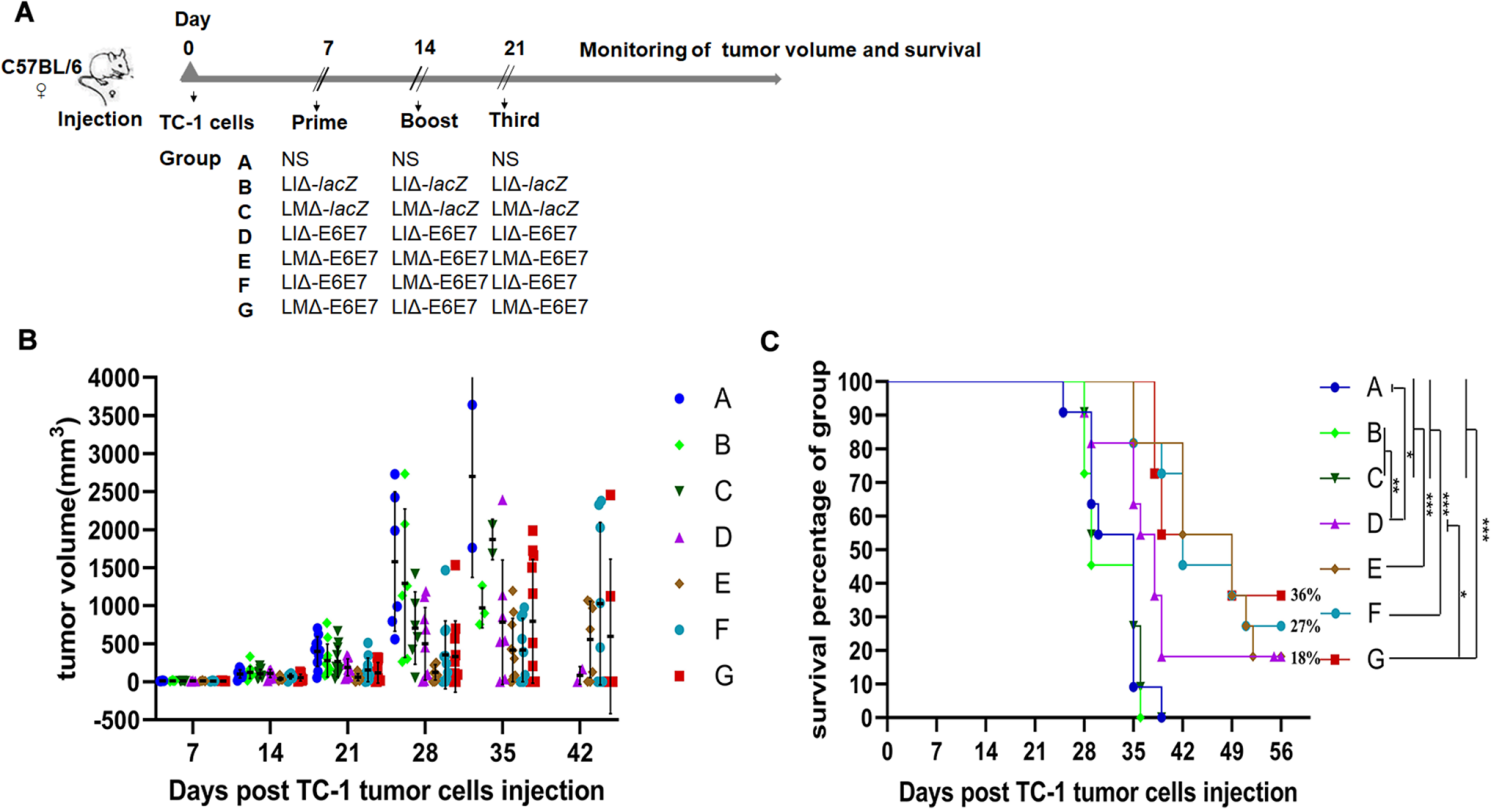
The tumor size and group survival curves of tumor-bearing mice after treated with different recombinant strains. Tumor model were established by subcutaneously injecting with 1 × 10^5^ TC-1 cells into the right flanks of C57BL/6J mice on day 0. (**A**) The figure showed the design of animal groups and the time schedule of treatment strategies (group A–G, 11 mice/group). On day 14 and 21, mice were boost immunized with specific strain or NS according to group design. The tumor size and survival were monitored after the primary immunization. (**B**, **C**) The change of tumor volume of each mouse in groups at different time points was presented, and the survival curve of mice in different group was compared. Data in this figure were representative of three independent experiments. ***P < 0.001, **P < 0.01, *P < 0.05.

To assess the antitumor effect of the recombinant strains, tumor-bearing mice were intravenously injected three times with the recombinant strains, vector strains or NS at weekly intervals. To compare the antitumor effect of different immunization strategies^17^, the mice were divided into several groups and received different treatments. As shown in Fig. 5, compared with control treatment, the recombinant strains significantly reduced the tumor sizes or slowed the growth of the tumors. Evaluation of mouse survival showed that the vaccinated mice in the therapeutic groups achieved longer survival time than the mice in the control group, and the survival rate of the combination therapy group LMΔ-E6E7 → LIΔ-E6E7 → LMΔ-E6E7 (G) was significantly longer than that of the single strain group (LIΔ-E6E7 (× 3)) (P < 0.05). At the end of the 56-day observation period, all the mice in the control and vector groups were died. Among the treatment groups, except the group D, where there were both tumor-bearing mice and cured mice survived, the other treatment groups were only had the cured mice survived. The survival rates of the treatment groups were 18% in group LIΔ-E6E7 (× 3) (D), 18% in group LMΔ-E6E7 (× 3) (E), 27% in group LIΔ-E6E7 → LMΔ-E6E7 → LIΔ-E6E7 (F) and 36% in group LMΔ-E6E7 → LIΔ-E6E7 → LMΔ-E6E7 (G), respectively. Moreover, the cure rates were 9% in group D, 18% in group E, 27% in group F and 36% in group G. In groups E, F and G, mice were cured two or three weeks after the first immunization, and mice in group D were cured in the fourth week after the beginning of immunotherapy. Among the single strain prime-boost immunotherapy groups (those treated with one strain repeatedly), the LMΔ-E6E7 group (E) showed better therapeutic efficacy than the LIΔ-E6E7 group (D). In addition, prime-boost immunotherapy with the two strains (alternate treatment with both strains) seemed to prove better therapeutic efficacy than a single strain prime-boost, which enhanced the survival rate of the mice. Among the different immunization regimens, LMΔ-E6E7 → LIΔ-E6E7 → LMΔ-E6E7 (G) showed the most notable antitumor efficacy.

### ***Recombinant strains decrease the proportion of Treg cells and enhance the proportion of CD8***^+^***T cells in the tumor microenvironment***

**Figure 6:**
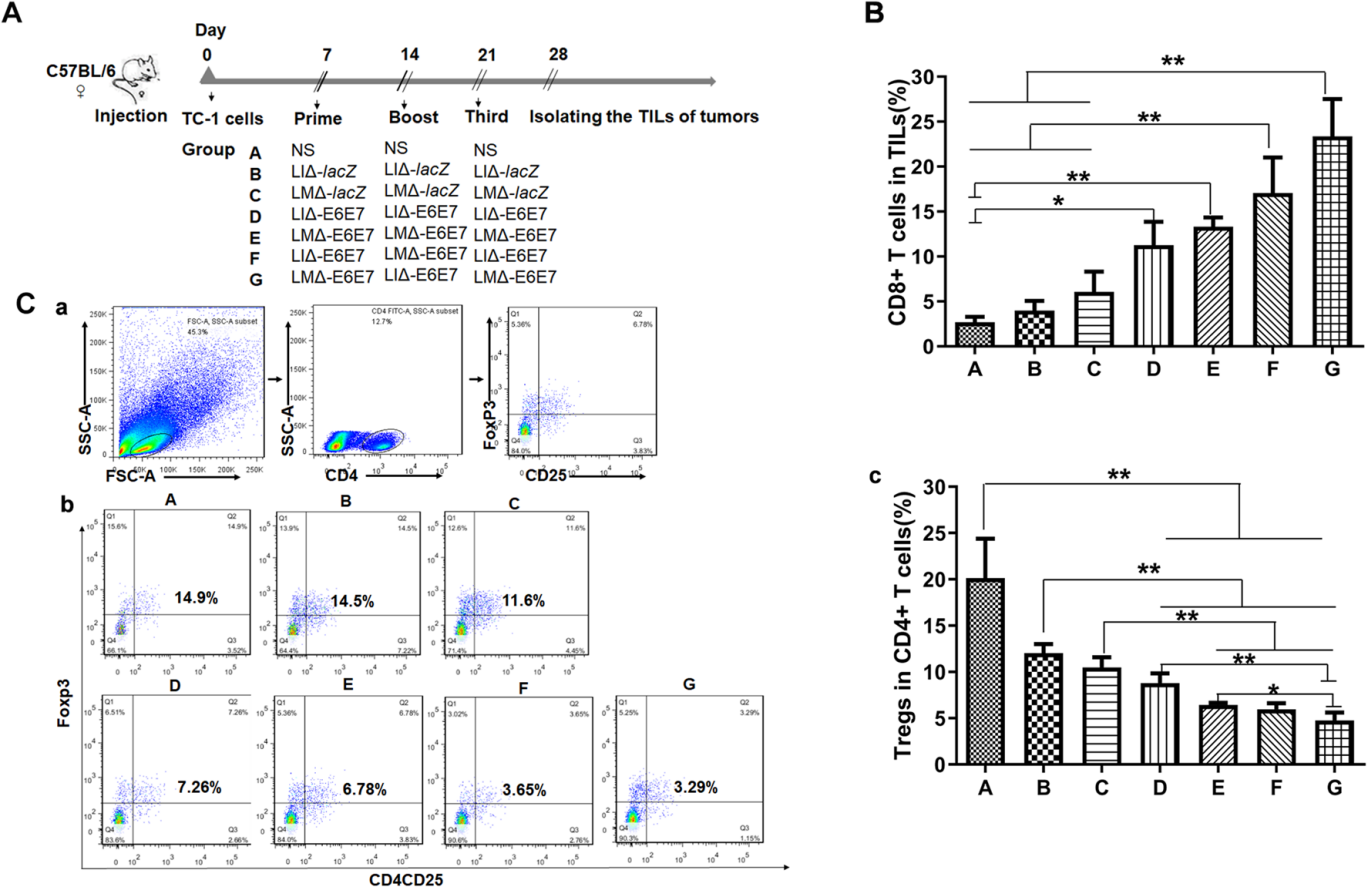
The percentage of Tregs and CD8^+^ T cells in TILs after immunotherapy. As (**A**) showed, TILs were isolated from tumors of mice in different groups (5 mice/group) at 7 days after the final vaccination. (**B**) Comparison of the percentage of CD3^+^CD8^+^ T cells in tumors of each group. (**C**) The percentage of Tregs in tumors. (a) Flow gate strategy. (b) Flow cytometry analysis of the frequency of CD4 + CD25 + Foxp3 + Tregs in tumors from mice of each group. (c) Comparison of the percentage of CD4^+^ CD25^+^ Foxp3^+^ Tregs of each group. Data in this figure are representative of three independent experiments. *P < 0.05, **P < 0.01.

Tregs in the tumor microenvironment play an important role in limiting antitumor immunity. Thus, the specific depletion of Tregs is sufficient for preventing tumor growth and prolonging survival time in mice^18^. The tumor infiltrating lymphocytes (TILs) isolated from the tumors were stained with CD4, CD25 and Foxp3 antibodies, and the stained cells were analyzed by flow cytometry. The results are presented in Fig. 6C. Comparing the results of the different groups (Fig. 6A) showed that the percentages of Tregs in therapeutic groups D, E, F and G were significantly lower than those in corresponding control groups A, B and C (Fig. 6C(c)). Moreover, the Treg percentage in mouse tumors of the two-strain prime-boost group G was lower than those in the single strain prime-boost groups. These results suggested that the recombinant strains significantly reduced the percentage of Tregs in the tumor environment. Combination immunotherapy with two strains was more effective at decreasing the proportion of Treg cells, especially the LMΔ-E6E7 → LIΔ-E6E7 → LMΔ-E6E7 combination.

The percentage of CD8^+^ T cells in TILs was also analyzed by flow cytometry. The percentages of CD8^+^ T cells in the therapeutic groups were significantly higher than those in the vector and naïve control groups (Fig. 6B). Similarly, the prime-boost immunotherapy with two strains induced a higher percentage of CD8^+^ T cells in TILs than prime-boost immunization with a single strain.

## Discussion

HPV infection and associated diseases continue to impose a heavy burden worldwide. It is known now that HPV serves as the etiological factor and biological carcinogen for cervical cancer. Progressive cervical cancer is the most incurable cancer among in women^19^. In recent years, immunotherapy for the cervical cancer has received a great deal of attention, and exciting developments have been made, especially related to therapeutic vaccines. Among the proteins encoded by HPV, E6 and E7 are the best target proteins for immunotherapy due to their carcinogenic role and constitutive expression in infected cells. In this study, a fusion protein containing the shuffled domains of HPV-16 E6 and E7 proteins was selected as a tumor-specific antigen. In this fusion protein, the amino (N-) and carboxyl-terminal (C-terminal) domains of the HPV-16 E6 and E7 proteins were alternatively placed, and an overlapping region of 16 amino acids at the junction sites was designed to minimize the loss of potential T-cell recognized epitopes^20^. The arrangement of the proteins could prevent the homodimerization of the E6 and E7 regions of the fusion protein, which is critical for their binding and for the degradation of the tumor suppressor proteins p53 and pRb. The HPV DNA vaccine (GX-188E) which expresses the fusion protein of that design, was shown to induce a E6/E7-specific T-cell response in all nine cervical intraepithelial neoplasia 3 (CIN3) patients. Moreover, the fusion protein was unable to degrade the p53 and pRb proteins as expected^20^. The above results indicate the effectiveness and safety of the selected antigen gene.

*Listeria* replicates and expresses heterologous recombinant proteins in the cytosol, causing both MHC I and MHC II antigen presentation; therefore, it is regarded as a promising live vaccine vector. A promising *Listeria*-based vaccine, Lm-LLO-E7 (also referred to as ADXS11-001) was designed by fusing HPV16 E7 with listeriolysin O (LLO)^21^. Clinical trails on patients with HPV associated cancers are currently ongoing. However, while the *Listeria* vector is an effective antigen delivery system, its safety must be seriously considered to avoid causing a safety risk to immunocompromised individuals. To data, several types of attenuated LM strains have been used in vaccine development. A previous study indicated that the LM attenuated strain with deletions of two major virulence genes, *actA* and *plcB*, is a safe vector for clinical application^22^. In studies by Yanyan Jia, an attenuated LM strain with deletions of *actA* and *plcB* was employed as a vaccine vector to deliver HPV16 E7. This highly attenuated vaccine caused little inflammatory injury in the spleen and liver and suppressed tumor growth in the mice^23^. Unlike previous studies, we innovatively used two *Listeria* strains as vaccine vectors for the delivery of the fusion antigen E6E7 and compared the antitumor effects between single-strain and two-strain prime-boost immunotherapy. LI is similar to LM in that it has a similar intracellular life cycle. In addition, it is nonpathogenic to humans, so it is superior to LM in terms of safety. Previous studies have shown that recombinant attenuated LI strains expressing *Mycobacterium tuberculosis* antigens are effective at inducing specific CD8^+^ T cell responses in mouse models^11^, demonstrating the potential of LI as a vaccine vector. The results of the present study suggested that both recombinant strains were able to induce tumor regression in the mice and that two-strain prime-boost immunotherapy exhibited the best therapeutic efficacy in terms of its effects on reducing tumor size and prolonging survival time.

The selected shuffled HPV-16 *E6E7* gene was integrated into the genome of the two attenuated *Listeria* strains by homologous exchange in this study. One of the recombinant strains, LIΔ-E6E7 stably expressed and secreted the targeted antigen protein in vitro. However, the target protein secreted by LMΔ-E6E7 was not detected in medium when cultured in vitro. Even though increasing the supernatant volume, the protein amount was still under the detection level. The difference in the PrfA between LM and LI may explain this phenomenon. In both recombinant strains, the expression of the fusion protein was promoted by the LM hly promoter (Phly). PrfA regulates the promoter *Phly* in listerial intracellular parasitism and activates the transcription of the related genes by specifically binding a palindromic promoter element of the canonical sequence tTAACanntGTtAa (PrfA box)^24^. A previous study indicated that LM PrfA’s regulatory function is amplified in the host cell cytosol but inhibited in its environmental habitat^24^. In another study by Mauder et al., the LI PrfA protein (PrfA_Li_) showed higher binding affinities to PrfA box than the LM PrfA protein (PrfA_Lm_) in vitro^25^. Therefore, the difference in PrfA may be the reason for the different expression levels between these two recombinant strains in vitro. Then we assessed the target protein expression within bacteria-infected cells to infer the expression status in vivo. The subsequent results showed that the fusion protein was successfully expressed at a similar level in LMΔ-E6E7-infected cells as LIΔ-E6E7-infected cells.

The success of cancer immunotherapy primarily relies on the induction of cellular immunity against tumor-specific antigen. IFN-γ activates macrophages, induces MHC II expression and indicates Th1 responses. IL-2 stimulates the differentiation of regulatory T cells and promotes T cell growth. TNF-α induces fever, apoptotic cell death, cachexia, inflammation and inhibition of tumorigenesis^26^. In our study, the percentages of IFN-γ-producing CD4^+^ and CD8^+^ T cells in the recombinant strain-immunized groups were significantly higher than those in the control groups. The percentages of TNF- or IL-2-producing CD4 + T and CD8 + T cells induced by LMΔ-E6E7 immunization were slightly higher than that of LIΔ-E6E7. Our results showed that both strains induced CD4^+^ T and CD8^+^ T cell responses and mainly elicited IFN-γ secretion. IFN-γ is a crucial cytokine in Th1 cell-mediated immunity against tumor cell proliferation^27^. The cell-mediated response triggered by LMΔ-E6E7 was clearly stronger than that triggered by LIΔ-E6E7. This finding may be associated with the bacterial load and survival time in the spleen, the important site for immune responses. After the mice were intravenously immunized, both recombinant *Listeria* strains invaded and localized to the liver and spleen. More LI than LM invaded the liver on 1 dpi, and LM showed a longer survival time in the spleen than LI. The difference in internalin B (InlB) protein and LLO between LM and LI is the primary reason for this phenomenon. InlB, which binds specifically to receptors in hepatocytes, is important for bacterial colonization in the liver. Although InlB1 and InlB2 of LI, which are encoded by the *inlB-1*and *inlB-2* genes, are similar to InlB of LM, the combination of InlB1 and InlB2 has a higher affinity for hepatocytes^28,29^. However, the liver is an organ with immune tolerance, and the immunosuppressive microenvironment of the liver leads the adaptive immune cells in the liver are prone to tolerance^30^. There are a large number of macrophages residing in the liver that known as Kupffer cells. However, in general, the ability of Kupffer cells to activate the adaptive immune response is poor^31^. This property leads to the inability of the liver to induce a strong immune response to the pathogen. LLO plays an important role in helping LM escape from phagocytosis. Ivanolysin O (ILO) of LI shares nearly 80% homology sequence with that of LLO. Previous studies have shown that introducing the ILO coding gene into LLO-knock-out LM could cause the defective strain to proliferate normally and persist for several days in the liver but was unable to survive in the spleen^10^. Therefore, it was inferred that the weak ability of LI of invading in the spleen might be related to some properties of ILO.

Recently, heterologous prime-boost immunization protocols using different gene expression systems have been shown to be successful for protecting against diseases in preclinical and even clinical trials^17,32^. Compared with those induced by homologous immunization, immune responses induced by heterologous immunization are less affected by anti-vector immunity, since different vector systems are used alternatively. Ideally, heterologous immunization selectively boosts the immune response targeting the specific antigen and induces higher memory CD8^+^ T cell responses^33,34^. In a study by Lin et al., priming with an HPV16 E6/E7 DNA vaccine and follow up with recombinant *vaccinia/adenovirus* or with a HPV16E6/E7-expressing tumor cell-based vaccine boost induced higher antigen-specific CD8^+^ T cell immune responses than vaccination with each vaccine individually^35^. A previous study indicated that anti-vector immunity of the same listerial strain does occur^36^. To avoid anti-vector immunity, we innovatively used a combination strategy that employed both attenuated LM and LI strains as vaccine vectors and subjected tumor-bearing mice to a prime-boost strategy in our study. Although LI and LM both belong to the *Listeria* genus, the homology of these two species at the DNA level is only 75%, which guarantees relatively weak anti-vector immunity when immunization is boosted. Two-strain prime-boost immunization provided better therapeutic efficacy than single-strain prime boost in terms of its effects on tumor size and survival time. We speculated that the reason is the lower anti-vector immunity. In addition, immunization with LMΔ-E6E7 as a prime dose and follow up with another strain alternatively was the best immunotherapy regimen. We speculated that prime immunization with LM induced stronger cell-mediated immune responses after inoculation thus effectively inhibiting the progression of the tumor before booster immunization.

The previous studies have showed that the antitumor mechanism of listeria-based therapeutic vaccines might be related to the reduced percentage of Tregs and CD8^+^ T cell infiltration in the tumor microenvironment^37,38^. Tregs have an adverse effect on antitumor immunity in many tumors^39^, including cervical cancer^40^. CD8^+^ T cells may participate in specific antitumor immune responses at tumor local sites^38^. Therefore, we detected the number of Tregs and CD8^+^ T cells from TILs in different treatment groups. We found that compared with the control, both vaccines enhanced the proportion of CD8^+^ T cells and reduced the proportion of Tregs in the tumor microenvironment, and LMΔ-E6E7 seemed more effective. In comparison with single-strain immunotherapy, two-strain combination immunotherapy exhibited better antitumor effects and was accompanied by fewer Tregs and a higher CD8^+^ T percentage in the TILs. Our results were consistent with the results reported by Yang et al.^41^, which demonstrated that *Listeria*-based vaccines could break the immunotolerance balance in the tumor microenvironment by inducing antigen-specific cytotoxic lymphocytes (CTLs) to participate in the antitumor immune responses at tumor local sites and by reducing the number of Tregs. Zhisong Chen reported that the decrease in Treg number induced by an LM-based vaccine was related to the function of LLO. LLO could preferentially induce the expansion of CD4^+^ Foxp3^−^ T cells and CD8^+^ T cells, thus reducing the percentage of Tregs^42^.

In conclusion, both LMΔ-E6E7 and LIΔ-E6E7 are safe and effective against TC-1-induced subcutaneous tumors. They successfully induce regression of tumor growth and extend survival time in tumor-bearing mice. Two-strain prime-boost immunization showed better therapeutic efficacy than single-strain regimens. We observed that the therapeutic effect was accompanied by a change in the proportion of CD8^+^ T cells and Tregs in the intratumoral milieu. The two candidate vaccines both have potential for future clinical application, and we propose that a combination strategy including both strains via prime-boost treatment is more efficient.

## Materials and methods

### Plasmids and bacterial strains

The plasmids and bacterial strains used in this paper are listed in Table 1.

**Table 1:** Plasmids and bacteria strains used in the present study.

Plasmids and bacteria	Relevant characteristics	Origin or reference
pCW154-E6E7	Derived from pCW154^16^, containing HPV-16 E6E7* coding gene cassette	This lab reserved
pCW702-E6E7	Derived from pCW154^16^, replaced LI homologous sequences with LM homologous sequences, containing HPV-16 E6E7* coding gene cassette	This lab reserved
LIΔ*actAplcB-lacZ*	Recombinant LI, lacking of *actA* and *plcB,* carrying *lacZ* gene in the genome	This lab reserved^16^
LMΔ*actAplcB-lacZ*	Recombinant LM, lacking of *actA* and *plcB,* carrying *lacZ* gene in the genome	This lab reserved^43^
LIΔ*actAplcB-*E6E7	Recombinant LI*,* lacking of *actA* and *plcB*, carrying HPV-16 E6E7* gene in the genome	This work
LMΔ*actAplcB-*E6E7	Recombinant LM*,* lacking of *actA* and *plcB*, carrying HPV-16 E6E7* gene in the genome	This work

E6E7*, the arrangement of *E6* and *E7* genes was intentionally shuffled, by alternatively placing the N- and C-terminal domains of the two genes, but with an overlapping region of 16 amino acids^17^.

### Mice and cell lines

The female C57BL/6J mice aged 6–8 weeks were purchased from the Institute of Laboratory Animals of Sichuan Academy of Medical Sciences & Sichuan Provincial People’s Hospital. The mice were maintained under specific-pathogen-free condition throughout the experiments at the Animal Center of the School of Public Health, Sichuan University. The mouse experiments were performed in accordance with the guidelines of the Animal Care and Use Committee of Sichuan University. The experimental protocols were approved by the Animal Care and Use Committee of Sichuan University. The animal study was performed in compliance with the ARRIVE guidelines.

The TC-1 cell line^44^, which was generated by the transformation of primary lung epithelial cells from C57BL/6J mice with HPV-16 E6 and E7 and an activated ras oncogene, was purchased from Beijing YZJ Biotechnology Company (Beijing, China). The cell line was cultured in RPMI 1640 supplemented with 10% fetal bovine serum, 100 U/mL penicillin and 100 μg/mL streptomycin at 37 °C under 5% CO_2_.

RAW264.7 murine macrophage-like cell was maintained in our laboratory. The cells were cultured in DMEM supplemented with 10% fetal bovine serum at 37 °C under 5% CO_2_.

### Construction of recombinant *Listeria* strains and antigenic protein expression

**Figure 7:**
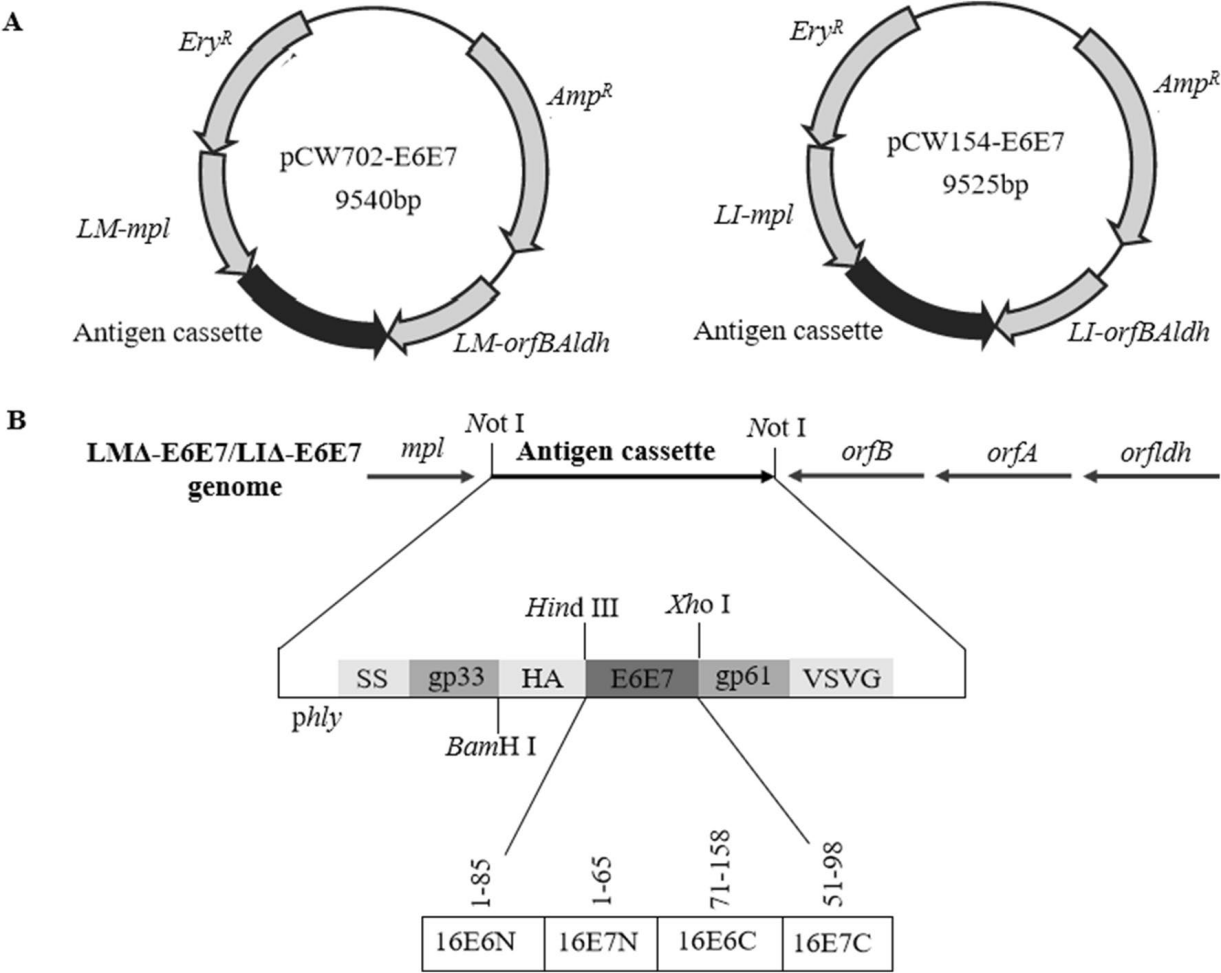
Schematic diagrams of targeting plasmids and the characterization of the recombinant strains LMΔ-E6E7 and LIΔ-E6E7. (**A**) Schematic diagrams of targeting plasmids pCW702-E6E7 and pCW154-E6E7. *Ery*^*R*^ erythromycin resistance gene, *Amp*^*R*^ ampicillin resistance gene. (**B**) The genome of the recombinant strains. The antigen cassette was integrated into LMΔ or LIΔ. p*hly*: LM *hly* promoter; SS: secretory signal sequence from the LM *hly* gene; gp33: the CD8^+^ T cell immunodominant epitope from lymphocytic choriomeningitis virus (LCMV) (gp33-41: KAVYNFATM); HA: HA Western blot tag (YPYDVPDYA); gp61: CD4^+^ T cell immunodominant epitope from LCMV (gp61-80: GLKGPDIYKGVYQFKSVEFD); VSV-G: VSV-G Western blot tag (YTDIEMNRLGK). The *E6E7* fusion gene was designed by inserting shuffled overlapping N- and C-terminal domains of *E6* and *E7* genes of HPV16. The abbreviation of viral domains is according with the gene and the domain, for example, 16E6N represents the N-terminal domain of HPV16 E6.

Two recombinant *Listeria* strains were constructed using the *Listeria* genome site-specific integration and expression system as described previously^16^ (Fig. 7). In brief, two targeting plasmids pCW702-E6E7 and pCW154-E6E7 were constructed first according to the methods described previously^16^, and they contained homologous *Listeria* sequences and a synthetic codon-optimized E6E7 gene that was shuffled by alternatively placing the coding region corresponding to the N terminal and C terminal domains of E6 and E7 proteins (Fig. 7)^20^. The plasmids pCW702-E6E7 and pCW154-E6E7 were electroporated into LMΔ*actAplcB-lacZ* (LMΔ*-lacZ*) and LIΔ*actAplcB-lacZ* (LIΔ*-lacZ*), respectively. The transformants were then cultured according to the method described by Lin et al.^11^. The desired recombinants, LMΔ-E6E7 and LIΔ-E6E7, were identified as erythromycin-sensitive white colonies, and were confirmed by genome PCR with the following primers and sequencing. *E6E7* (F): 5′-AACGTACAGCAATGTTCCA-3′, *E6E7* (R): 5′-GCCTCGAGTTATCATGGTTT-3′; *Ery* (F): 5′-GTCGACGATTCACAAAAAATAGGC-3′, *Ery* (R): 5′-ACTAGTCCCGGGGCGAATTG-3′; *LI-actA* (F): 5′-GAAGCTAAAAGTGCAAATGTCCC-3′, *LI-actA* (R): 5′-ATTTCTTTAATACTGCGTTTGGGG-3′; and *LM-actA* (F): 5′-GCTATAAATGAAGAGGCTTCAGG-3′, *LM-actA* (R): 5′-CTCTTAAATCAGCTAGGCGATC-3′.

LIΔ-E6E7 total protein in 35 mL supernatant and from the total cellular lysate obtained by centrifugation of 6 mL of recombinant strain culture were collected by trichloroacetic acid precipitation according to the method described by Lin et al.^11^. LM-E6E7 total protein was collected from the total cellular lysate obtained by centrifugation of 300 mL culture to measure intracellular target protein expression or by centrifugation of 300 mL supernatant for extracellular target protein assay. The protein samples were separated on an 8% SDS gel and then transferred onto a PVDF membrane. The membrane was incubated with anti-Influenza Hemagglutinin (anti-HA) monoclonal primary antibody (1:5000) (Sigma-Aldrich, USA) and followed by incubation with horseradish peroxidase-conjugated secondary antibodies (1:1000) (Beyotime Institute of Biotechnology, Shanghai, China). The signals were detected using Super Signal West Pico (Thermo Scientific, USA).

### Antigenic protein expression in RAW264.7 cells

The recombinant strains were cultured in BHI broth to mid-logarithmic phase, and then the bacteria were centrifuged, collected and diluted with DMEM supplemented with 10% fetal bovine serum.

The RAW264.7 cells were grown in 12-well plates for 24 h and then infected with recombinant strains at an MOI of 100:1 for 1 h, at 37 °C under 5% CO_2_. The cells were washed with PBS and treated with DMEM supplemented with 200 μg/mL gentamicin for 1 h to kill extracellular bacteria. The cells were cultured for 6 h in DMEM supplemented with 20 μg/mL gentamicin before being lysed with RIPA buffer (Solarbio, China). The supernatant was collected after centrifuging the cell lysate, and the target antigenic protein levels was measured by Western blotting.

### Determining the of 50% lethal doses of each recombinant bacterial strain

The recombinant strains were cultured to mid-logarithmic phase then centrifuged to collect the bacteria, and the bacteria were diluted in NS. C57BL/6J mice were randomly divided into three groups. The groups of mice were injected intravenously with various doses of bacteria (10^7^, 10^8^ or 10^9^ CFUs per mouse), and mortality was calculated continuously for 10 days. The LD_50_ value was determined using the improved Karber method^45^.

### Bacterial loads of recombinant bacterial strains in infected mice

The C57BL/6J mice in each group were injected intravenously with a 0.1 × LD_50_ dose of each strain (4 × 10^7^ CFUs of LIΔ-E6E7; 1.3 × 10^7^ CFUs of LMΔ-E6E7). The mice were sacrificed on 1, 2, 3, 5, 7, and 14 dpi. Liver and spleen were collected aseptically and homogenized in sterile buffer containing 0.1% Triton X-100. The homogenates were serially diluted tenfold with 0.1% Triton X-100 and plated on BHI agar plates. The colonies on the plates were counted after 24–48 h of growth at 37 °C.

### Detection of intracellular cytokines by flow cytometry

To analyze the immunogenicity of the recombinant strains, the mice were immunized intravenously (i.v.) with NS, 4 × 10^7^ CFUs of LIΔ-E6E7, 2 × 10^7^ CFUs of LIΔ*-lacZ*, 1.3 × 10^7^ CFUs of LMΔ-E6E7 or 5 × 10^6^ CFUs of LMΔ*-lacZ* (the dose was calculated as the 0.1 × LD_50_ of each strain). Intracellular cytokine staining was performed on 9 dpi, using freshly prepared single splenocyte suspensions. The splenocytes were stimulated for 5 h with or without mixed peptides (Sangon Biotech, Shanghai, China), including the CD8^+^ T cell immunodominant epitope from lymphocytic choriomeningitis virus (LCMV) (gp33–41: KAVYNFATM), the CD4^+^ T cell immunodominant epitope from LCMV (gp61-80: GLKGPDIYKGVYQFKSVEFD), HPV16 E7 49-57 (RAHYNIVTF), HPV16 E7 11-20 (YMLDLQPETT), HPV16 E7 86-93 (TLGIVCPI) and HPV16 E6 50-57 (YDFAFRDL). The cell surface antigens were stained with FITC-conjugated rat anti-mouse CD3 (17A2, Biolegend, USA), PerCP-conjugated rat anti-mouse CD4 (RM4-5, Biolegend, USA) and APC-Cy7-conjugated rat anti-mouse CD8a (53-6.7, BD Pharmingen, USA) for 30 min at 4 ℃. Then, the cells were permeabilized using the Cytofix/Cytoperm kit (BD Pharmingen, USA) for 20 min at 4 °C. Intracellular cytokines were stained with PE-conjugated rat anti-mouse IFN-γ (XMG1.2, Biolegend, USA), PE-Cy7-conjugated rat anti-mouse TNF-a (MP6-XT22, Bioligand, USA), and APC-conjugated rat anti-mouse IL-2 (JES6-5H4, Biolegend, USA) for 45 min at 4 °C. After being stained with surface and intracellular antibodies, the cells were fixed in 2% (wt/vol) paraformaldehyde-PBS, and were analyzed on a BD FACSVerse flow cytometer.

### Establishment of a tumor model and evaluation of the therapeutic efficacy of the recombinant strains

TC-1 cells (1 × 10^5^) were subcutaneously injected into the right flanks of C57BL/6J mice. After 7 days, tumor-bearing mice were randomly distributed into seven groups, including the control group, which received NS (× 3) (A); vector groups, which received LIΔ*-lacZ* (× 3) (B) or LMΔ*-lacZ* (× 3) (C); and treatment groups, which received LIΔ-E6E7 (× 3) (D), LMΔ-E6E7 (× 3) (E), LIΔ-E6E7 → LMΔ-E6E7 → LIΔ-E6E7 (F) or LMΔ-E6E7 → LIΔ-E6E7 → LMΔ-E6E7 (G). All the groups were immunized intravenously with 0.1 × LD_50_ of the appropriate strain in an injection volume of 100 μL or 100 μL NS as a placebo on days 7, 14, and 21 after tumor cells injection according to the different treatment strategies. For example, the mice in group E were primarily immunized with LMΔ-E6E7 on day 7 and boost-immunized with LMΔ-E6E7 on days 14 and 21. The shortest and longest diameters of the tumor were measured with calipers, and mouse survival was monitored daily for 56 days. The mice were sacrificed when the longest tumor diameter reached 20 mm or the experimental observation period ended.

### Flow cytometry analysis of Tregs in TILs

Seven days after administration of the third immunotherapy, 0.5 g of tumor tissue was excised, cut into 1–2 mm pieces and digested in RPMI 1640 medium supplemented with 5% fetal bovine serum, 1 mg/mL collagenase type I (Sigma, USA) and 60 U/mL DNase I (Sigma, USA) for 35 min at 37 °C. Then the cell suspensions were filtered through 70 µm cell strainers, and the TILs were collected using a mouse tumor-infiltrating lymphocyte isolated tissue fluid kit (TBD science, Tianjin, China) according to the manufacturer’s recommended protocol. Tregs in the TILs were identified by flow cytometry after being stained with FITC-conjugated rat anti-mouse CD4 (RM4-5, eBioscience, San Diego, CA, USA), APC-conjugated rat anti-mouse CD25 (PC61.5, eBioscience, San Diego, CA, USA) and PE-conjugated rat anti-mouse Foxp3 (FJK-16 s, eBioscience, San Diego, CA, USA).

### Flow cytometer analysis of CD8^+^ T cells in TILs

TILs were obtained as described above. The cells were stained with FITC-conjugated rat anti-mouse CD3 and APC-Cy7-conjugated rat anti-mouse CD8a for 30 min at 4 °C. Then, the stained cells were assayed with a flow cytometer.

### Statistical analysis

All the statistical analyses were performed using SPSS ver. 19.0 (Chicago, IL, USA) and GraphPad Prism 5.0. One-way analysis of variance (ANOVA) followed by Dunn’s multiple comparison test or rank-sum test was used to determine the significance of the differences between groups. Kaplan–Meier analyses were used to estimate the survival curves of different groups, and significance was evaluated using the log-rank test. All the results were considered statistically significant at *P* < 0.05.

## Supplementary Information

Supplementary Figure.

## Data Availability

The datasets analyzed during the current study are available from the corresponding author on reasonable request.

## Code availability

*Accession codes* pCW154-E6E7: NCBI accession no.MT470408. pCW702-E6E7: NCBI accession no.MT470409.
