# Supplementary material for: Combination immunotherapy with two attenuated Listeria strains carrying shuffled HPV-16 E6E7 protein causes tumor regression in a mouse tumor model
Source: Sci Rep. 2021 Jun 28;11:13404. doi: 10.1038/s41598-021-92875-9 (PMC8238941; doi:10.1038/s41598-021-92875-9)
Supplement: Supplementary file 1 — Supplementary Figure. [file 41598_2021_92875_MOESM1_ESM.pdf]

Combination immunotherapy with two attenuated Listeria strains carrying shuffled HPV-16 E6E7 protein cause tumor regression in a tumor mouse model

Lin Su [a, b#], Yunwen Zhang [a, b#], Xiang Zhang [a, b], Ting Liu [a, b], Sijing Liu [a, b], Yongyu Li [a, b], Mingjuan Jiang [a, b], Tian Tang [a, b], Haiqian Shen[c *], Chuan Wang [a, b *]

[a] West China School of Public Health and West China Fourth Hospital, Sichuan University, Chengdu, P. R. China, [b] Food Safety Monitoring and Risk Assessment Key Laboratory of Sichuan Province, West China School of Public Health, Sichuan University, Chengdu, P. R. China, [c] Nanjing Sungyee Biotechnology Co., Ltd, Nanjing, P. R. China

*Correspondence: Chuan Wang, West China School of Public Health and West China Fourth Hospital, Sichuan University, 17#, Section 3, Renmin Nan Road, Chengdu, Sichuan, 610041, P. R. China, wangchuan@scu.edu.cn

Haiqian Shen, shen@sungyee.com

# These authors contributed equally to this work

Uncropped versions

Fig1.(A)

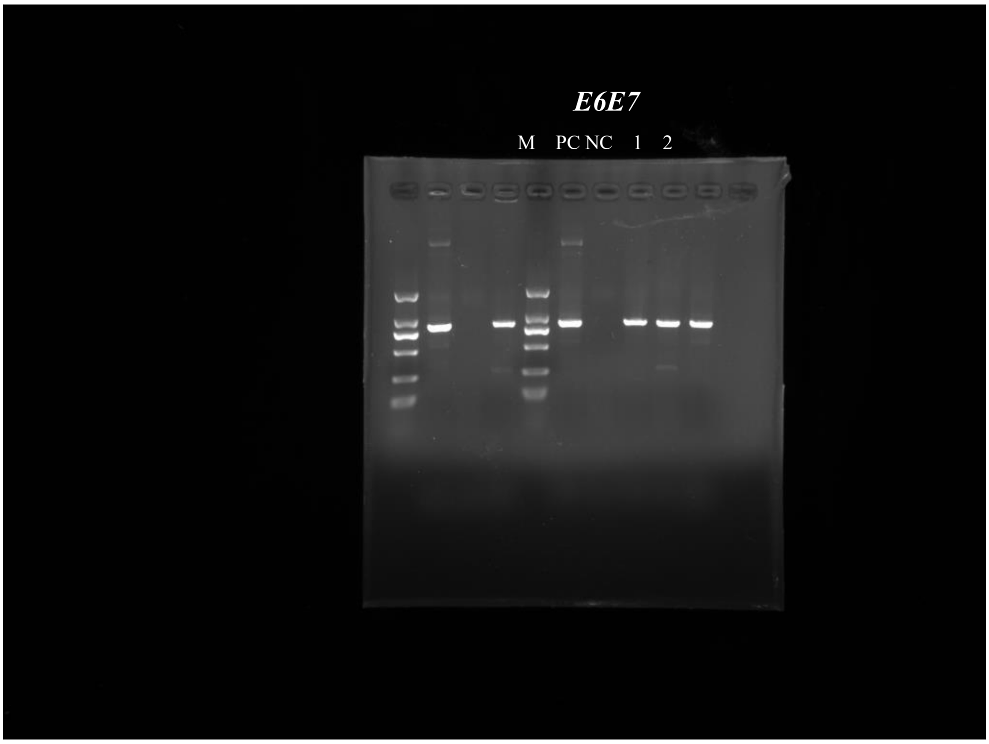

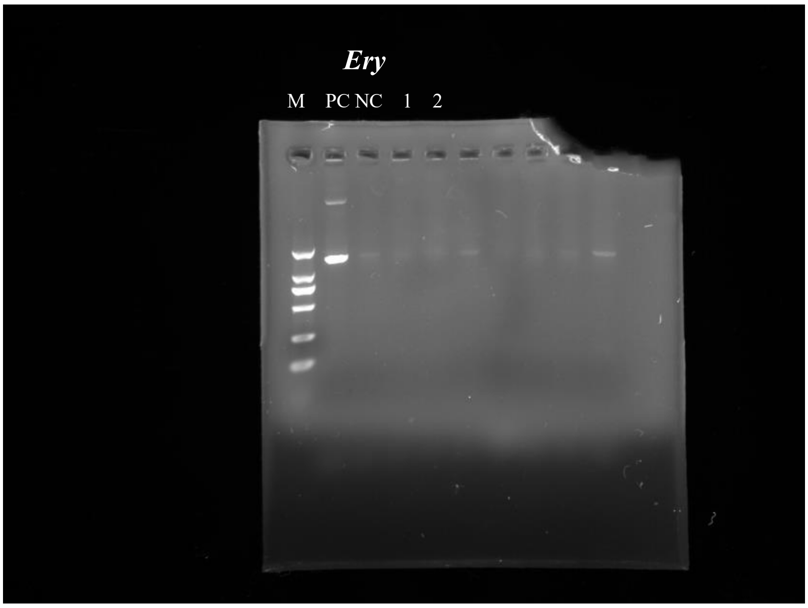

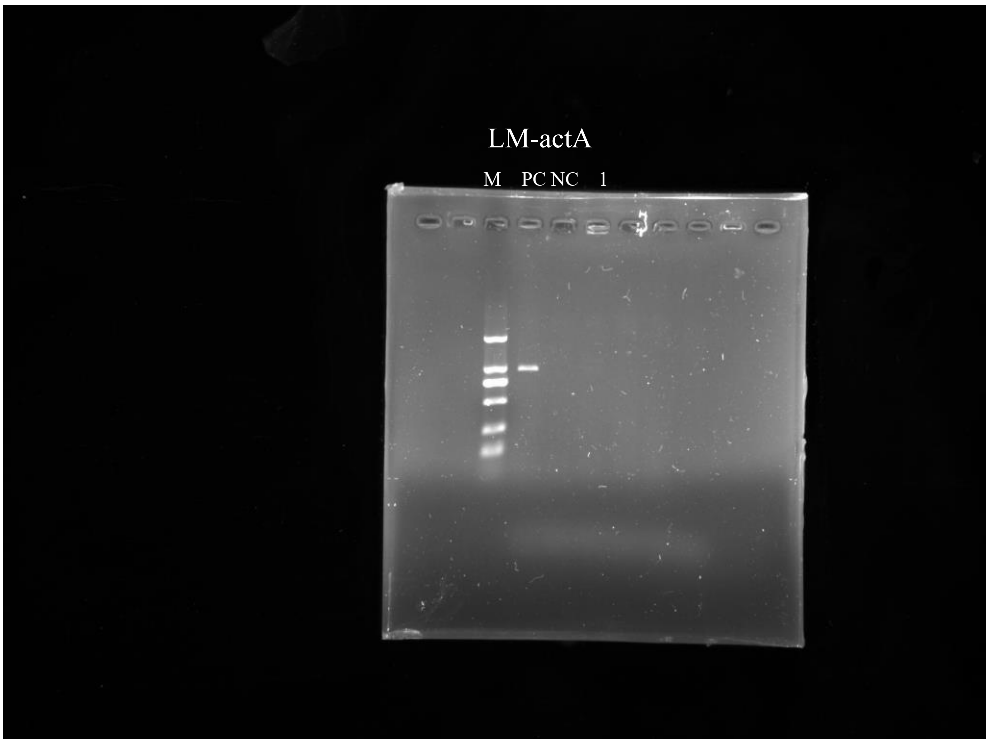

LI-*actA*

M PC NC 2

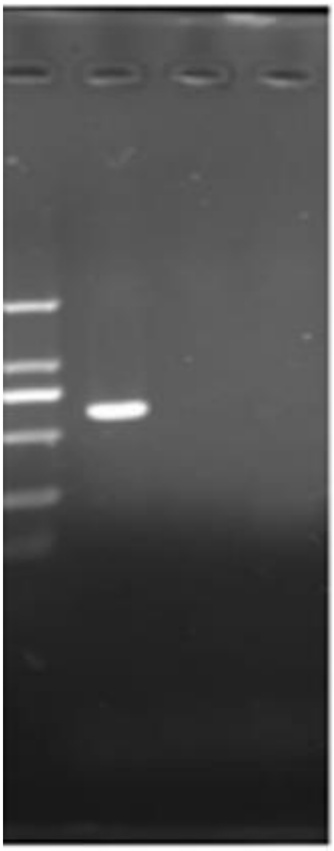

Fig1.(B)

LIΔ-E6E7:

Cell lysate Supernatant

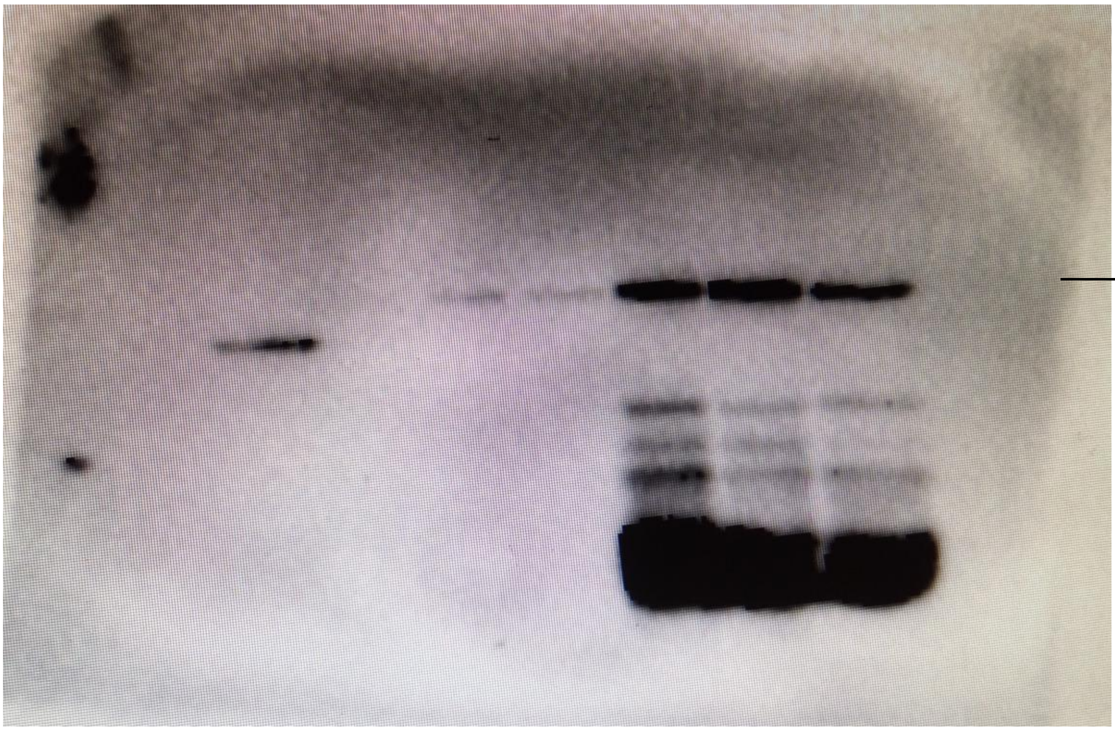

46kDa

(C)

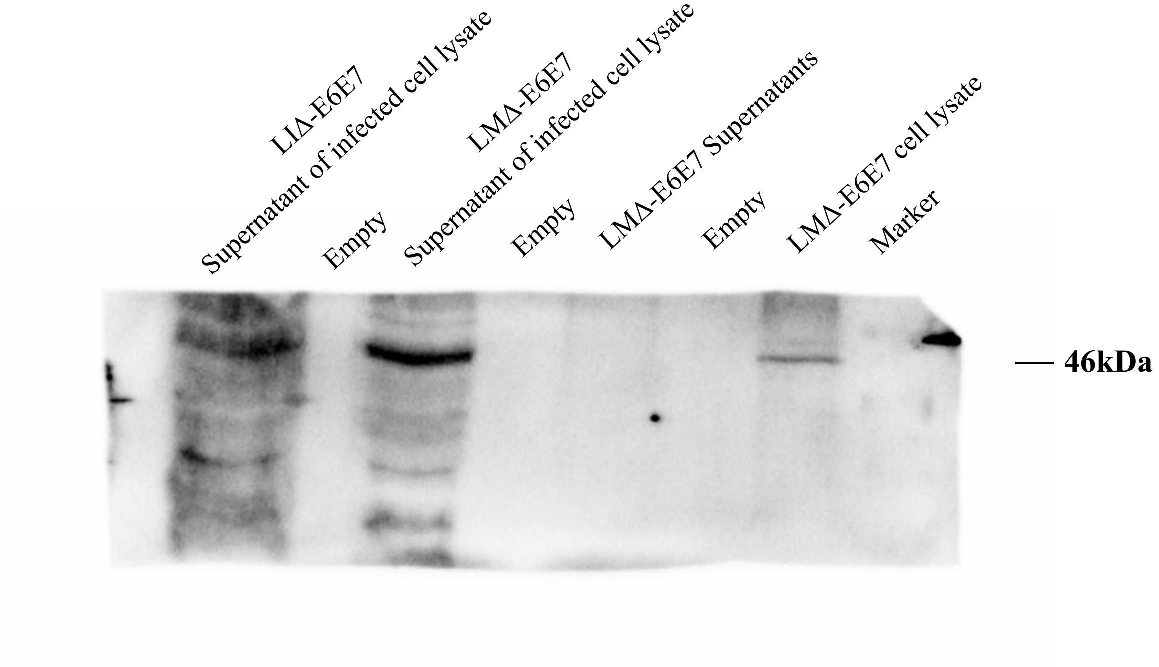
LIΔ-E6E7
Supernatant of infected cell lysate
Empty
LMΔ-E6E7
Supernatant of infected cell lysate
Empty
LMΔ-E6E7 Supernatants
Empty
LMΔ-E6E7 cell lysate
Marker
46kDa
